# The Retail Food Environment Index and its association with dietary patterns, body mass index, and socioeconomic position: A multilevel assessment in Mexico

**DOI:** 10.1371/journal.pgph.0003819

**Published:** 2024-10-10

**Authors:** Elisa Pineda, Jemima Stockton, Jennifer S. Mindell

**Affiliations:** 1 The George Institute for Global Health UK, London, United Kingdom; 2 School of Public Health, Imperial College London, London, United Kingdom; 3 Research Department of Epidemiology and Public Health, University College London, London, United Kingdom; Qatar University College of Medicine, QATAR

## Abstract

In Mexico, 75% of the population are affected by overweight or obesity, and the availability and affordability of high-calorie-dense foods and beverages are high. This study tested the association between the retail food environment index (RFEI), dietary patterns, body mass index (BMI), and socioeconomic position (SEP) in Mexico. Cross-sectional diet, health, and sociodemographic population-based secondary data analyses were conducted. The RFEI was calculated by dividing the total number of fast-food outlets and convenience stores by the total number of supermarkets and fruit and vegetable stores per census tract area. Associations between BMI, dietary patterns, SEP and the RFEI were tested using multilevel linear regression, including interactions of the RFEI with SEP, gender, and age. Living in neighbourhoods with a higher RFEI was associated with a 0.01kg/m^2^ higher BMI (β = 0.01, 95%CI: 0.0005, 0.02, p = 0.04), equivalent to a mean 0.046 weight gain for a 1.60m tall person per 10% higher RFEI. Unhealthy dietary patterns were more likely in neighbourhoods with a higher RFEI (β = 0.100, 95%CI: 0.03, 0.12, p = 0.001). Multilevel linear regression showed that lower SEP households had a higher RFEI compared to higher SEP households (β = 0.020, 95% CI: -0.006 to 0.04, p = 0.10). Generalised structural equation models revealed a graded relationship between RFEI and SEP, showing that lower SEP households were exposed to a higher RFEI (β = 0.060, 95% CI: 0.05 to 0.07, p < 0.001.) The study identified significant associations between higher proportions of fast-food outlets and convenience stores, higher BMI, and unhealthy dietary patterns. It was particularly evident that low-income populations are more likely to be exposed to obesogenic food environments.

## Introduction

Low- and middle-income countries have been greatly affected by unprecedented levels of obesity, non-communicable diseases, and by nutrition and food retail transitions [1–4]. In Mexico, in 2018, 75% of the population were affected by increased weight or obesity [4, 5]. Concurrently, ultra-processed food sales have continued to grow [6]. According to the 2019 Pan-American Health Organization (PAHO) report, Mexico had one of the highest sales of ultra-processed foods, with 522 kcal per capita/day [6]. The 2012 Health and National Survey (ENSANUT) in Mexico identified an average consumption of 163 L per capita/year of sugar-sweetened beverages (SSBs) [4, 7].

Pervasive ultra-processed food advertising and an increased exposure and accessibility to food outlets that offer affordable foods high in fat, sugar, and salt (HFSS) have modified the way we eat and increased our risk of obesity [4, 8, 9]. Americans now consume on average 500 more calories a day than they did in the 1970s [10]. Similarly in other countries, ultra-processed consumption continues to rise [11]. Differences in obesity rates across populations may indicate varying genetic susceptibilities to the food environment [4, 12]. However, the increase in the mean weight of the worldwide population in the last 30 years is likely to have been driven by food environment changes [4, 12–14].

The food environment refers to the built environment settings where food is available for acquisition by the population [4]. It may be influenced by policies, sociocultural norms, local conditions, marketing, and economic factors [4, 15]. These elements drive accessibility, affordability, convenience, and desirability, ultimately leading to food choice [4, 15–17]. Measures of the food environment have involved determining the density or proximity of food outlets. Alternatively, indices, such as the Retail Food Environment Index (RFEI), created by the California Center for Public Health Advocacy [18, 19], are important tools to visualize the food environment as a whole rather than looking at specific food outlets in isolation and to identify patterns which may explain variations in the food environment and their relationship with obesity.

Previous studies in Mexico have studied the density of specific food outlets and body mass index (BMI) [4, 6]. However, no studies have used tools such as the RFEI to assess associations between the food environment, diet, BMI, and socioeconomic position (SEP) in Mexico. In addition, no food retail regulations have been introduced at a national level in Mexico to reduce the availability of unhealthy foods and improve the availability and accessibility of healthy foods and beverages in food outlets [4]. Therefore, this study has aimed to advance the evidence by looking deeper into food environment patterns and analyse the association between the RFEI, BMI, dietary patterns, and SEP in urban areas of Mexico to inform policymaking regarding food environment regulation. We hypothesized that a low availability of food outlets offering unprocessed food and beverages, coupled with a high availability of stores offering calorie-dense foods and beverages, would be associated with unhealthy dietary patterns and higher BMIs.

## Methods

This study encompassed an analysis of secondary data from the national health and nutrition examination survey in Mexico to assess the RFEI and its association with dietary patterns, BMI, and SEP.

### Population sample

Dietary, sociodemographic, economic, health, and anthropometric variables were acquired from the 2012 Mexican National Survey of Health and Nutrition (ENSANUT), a health examination survey conducted in the homes of a random sample of the general population, based on residence [4, 20]. BMI was calculated in a previous study [4] and used as a measure of obesity in adults. The following selection criteria were applied: weight and height data from the 20+ (adults) and 15–19 years (adolescents) datasets were merged into a single database (n = 84,747). Individuals without valid weight (due to refusal, missing data, or faulty measurement) (n = 4,630) or height (n = 4,725) measurements were excluded, resulting in a BMI dataset of n = 76,113. Those younger than 18 years of age (n = 36,485) were excluded, creating a 18+ BMI dataset of n = 39,628 individuals. Women who were pregnant, breastfeeding, or did not respond to the relevant question, were also excluded (n = 1,592), leaving a sample of n = 37,969 [4]. Participants with BMI values of >3 standard deviations from the mean (<15 kg/m^2^ and >58 kg/m^2^) were excluded (n = 67) to avoid the inclusion of individuals with possible underlying illnesses, eating disorders, or erroneous data and to ensure normal BMI distribution [4]. We then excluded individuals living in rural areas or lacking geographic measure (n = 24,251). After merging individual and geographic and dietary data, we retained an analytical sample of n = 13,718 individuals.

### Dietary, anthropometric, and sociodemographic variables

Variables which influenced the selection of individuals into the ENSANUT survey (urbanicity, healthcare service user, food assistance program participation) were used in the analytical models to account for selection-bias [4, 19]. The 2012 ENSANUT sample deliberately included a higher proportion of lower SEP households [20]. This approach aimed to effectively monitor how social and nutrition programs impact households that are most likely to benefit from monetary educational grants [20]. Participants’ BMI was classified as underweight: 15 to <18.5 kg/m^2^; healthy weight: ≥18.5 to <25 kg/m^2^; overweight: 25 to <30 kg/m^2^; and obese: ≥30 kg/m^2^ to 58 kg/m^2^ [4, 19].

Data on potential confounding variables were gathered from the 2012 ENSANUT survey [4, 20], encompassing factors like age, gender, SEP, physical activity, car ownership, neighbourhood deprivation level, participation in a food assistance programme, and registration with a healthcare service provider [4]. Of these variables, physical activity and car ownership, and dietary data, were only asked in a module administered only to a subsample [20].

### Food environment data and classification

Food retail data were obtained in a previous study [4] from the 2014 Economic census from the National Institute of Statistics and Geography (INEGI) [21]. Urban census tract areas (CTA) were used as a proxy for neighbourhoods [4, 6].

The utilization of urban areas as a focal point in our investigation is rooted in their relevance to the food environment and its impact on dietary patterns and BMI [4]. Urban areas often exhibit distinctive food landscapes characterized by varying levels of food outlet density and accessibility, influencing the dietary choices and health outcomes of their residents [4]. Therefore, our study focuses on these urban environments to discern the intricate relationships between the retail food environment, dietary patterns, and BMI, with a specific emphasis on their implications for socioeconomically disadvantaged populations.

Food outlet classification was undertaken in a previous study [4]. The classification was undertaken considering the economic census INEGI conducted in 2014, employing digital georeferencing to document various establishment characteristics, including food retailers [4, 6, 21]. INEGI designates urban CTAs as geographic areas characterized by a population size of ≥2,500 inhabitants and contain 1 to 50 blocks, which are delimited by streets, avenues, or identifiable pathways [21]. This classification encompasses zones primarily utilized for habitation, industrial activities, or commercial services [6, 21]. Urban CTAs differ from rural CTAs in that they are geographic areas with < 2,500 inhabitants and are delimited by natural features (e.g. rivers, streams, ravines), and cultural features (e.g. railroad tracks, power lines, highways, dirt roads, paths, and pipelines property boundaries). Additionally, metropolitan areas are defined as set of municipalities that share a high degree of SEP interaction with more than one million inhabitants. By adopting this definition, we aimed to ensure a standardized and reliable classification of urban areas, allowing for consistent analysis and interpretation of the study’s findings [4].

INEGI also utilized surveys and digital georeferencing to gather information on the type of outlet, food provided, number of employees (indicative of outlet size), website, telephone number, and location characteristics [4, 19].

Based on this dataset, INEGI classified food retailers into categories such as restaurants, supermarkets, fruit and vegetable stores, chain-type convenience stores, and non-chain-type convenience stores [4, 6, 19, 21]. To refine our study’s food outlet classification, which is crucial to the exposure variable, we relied on INEGI’s data regarding outlet characteristics and the types of food they offered. Notably, our approach did not involve in-store assessments. When uncertainty arose about a food outlet’s classification, we assessed and categorized it based on the main food products sold and available on the outlet’s website and based on our previous study [4].

Specifically for this study, establishments, including informal and mobile food carts, specializing in items like pizzas, hamburgers, hotdogs, and fried chicken, were classified as fast-food outlets [4]. Those predominantly offering SSBs, and unhealthy snacks were designated as convenience stores. Based on the assumption that all fast-food outlets and convenience stores primarily sold SSBs, snacks, and ultra-processed foods [4]. Outlets featuring à la carte menus and sitting options were categorized as restaurants [4]. Mega-supermarkets and grocery stores, offering a broader range of food options, including fruits and vegetables, were classified as supermarkets [4], and outlets specializing in fruits and/or vegetables were categorized as fruit and vegetable stores [4, 6].

### Geocoding of individuals and food outlets

Geolocation of the 2012 ENSANUT participants was carried out to the centroid of each urban CTA using the geographic information systems software ArcGIS 10.2.2 (ESRI, Redlands, CA) [4, 22]. Spatial coordinates of food outlets were obtained from INEGI, 2014 [4, 21]. Using geographic information systems, data from the urban CTA maps were merged with the geolocation of food outlets and participant data which included health, nutritional and sociodemographic characteristics. Ground-truthing (i.e., verification of geographic data) was undertaken for our previous study [4, 19] in sample areas (S1 Text). This process consisted of verifying the accuracy of the food outlet database obtained from INEGI. The verification process checked the geolocation of all food outlets in the sample areas, their existence and type of food outlet [4, 23].

Ground-truthing was carried out in the city of Hermosillo in Mexico, to confirm the locations of various food outlets, such as fast-food outlets, convenience stores, supermarkets and fruit and vegetable stores. Hermosillo’s 346 urban CTAs were the focus, with nine areas randomly chosen from different cardinal directions. Food establishments were mapped using symbols based on their geolocation. Verification was conducted both on foot and by car, with photos taken to confirm the existence and precise locations of the outlets. These areas varied by SEP, influencing the types of food establishments present. The results showed an increase in supermarkets and convenience stores and a significant presence of informal food outlets not previously recorded in the INEGI database. Overall, 84% of the food outlets from the 2014 INEGI dataset matched the actual locations verified in 2016, while 16% showed discrepancies due to urban development changes. This temporal variation underscores the importance of caution when interpreting the association between the RFEI and health outcomes in Mexico.

### Retail Food Environment Index assessment

In this study, we first calculated the number of fast-food outlets, convenience stores, supermarkets and fruit and vegetable stores per CTA using ArcGIS 10.2.2 (ESRI, Redlands, CA). Then, the RFEI was calculated by dividing the total number of fast-food outlets and convenience stores by the total number of supermarkets and fruit and vegetable stores per CTA. A higher RFEI score represents a food environment with a larger number of fast-food outlets and convenience stores, which tend to have a higher offering of foods high in HFSS compared to supermarkets and fruit and vegetable stores, which tend to have a higher offering of fruits and vegetables and nutrient-dense foods. Descriptive statistical analyses were conducted to identify key RFEI metrics, including the minimum, maximum, and interquartile range (which comprised the first quartile Q1, the median Q2, and the third quartile Q3).

### Dietary patterns

Our approach to deriving dietary patterns followed the methodology outlined in our previous study [24]. Utilizing exploratory factor analysis (FA), we employed principal component analysis and varimax rotation as the extraction method. To ensure the appropriateness of FA to our data, we conducted the Kaiser-Meyer-Olkin test. The number of factors to retain was determined through the examination of a scree plot. Food items demonstrating factor loadings ≥0.30 were retained in the pattern. Factor loadings are coefficients that represent the relationship between observed variables (in this case, food groups) and the underlying latent factors (dietary patterns). They indicate the strength and direction of these relationships, with higher absolute values suggesting stronger associations. Factor scores, on the other hand, are estimated values for each individual, reflecting their adherence to the identified dietary patterns. These scores are used in subsequent analyses to explore the associations between dietary patterns and health outcomes such as BMI and SEP [24].

For dietary pattern computation we aggregated 140 food items from food frequency questionnaires [24]. We grouped food items based on nutritional content and common habitual consumption patterns in the study population, which represented 11% of the ENSAUT participant sample, resulting in an analytical sample of n = 1,572 [24]. Unhealthy dietary patterns were defined based on high factor loadings for food groups typically considered less healthy due to their high content of fats, sugars, and refined carbohydrates. Specifically, a higher factor score on the ’unhealthy dietary pattern’ indicated greater adherence to a diet rich in foods such as sausages, sugar-laden desserts, soda, fast-food items, and potato chips. Conversely, a higher factor score on the ’healthy dietary pattern’ indicated greater adherence to a diet rich in vegetables, fruits, and other nutrient-dense foods. Thus, a participant with a higher score on the ’unhealthy dietary pattern’ and/or a lower score on the ’healthy dietary pattern’ was considered to have a less healthy diet. The factor scores, derived from this process, were subsequently integrated into our regression analysis using SAS OnDemand for Academics (SAS Institute Inc., Cary, USA) [24, 25]. For a detailed breakdown of the specific foods corresponding to each category, please refer to S3 Text. Our classification of dietary patterns aligns with our previous publication [24] wherein Factor 1 was identified as a healthy pattern, Factor 2 as an unhealthy pattern, and Factor 3 as a carbohydrate-oriented dietary pattern (S3 Text).

### Retail Food Environment Index and socioeconomic position

To further understand the relationship of SEP with the RFEI and BMI, our analysis comprised: 1) a multilevel logistic regression to test the association of SEP (categorical outcome) with the RFEI (exposure), adjusted for age and gender; 2) a multilevel linear regression model to test the association between BMI (outcome) and the RFEI (exposure), stratifying models by SEP quintiles; 3) generalized structural equation modelling to test the association of SEP (outcome) with the RFEI (exposure); and 4) interaction tests between SEP and RFEI, as well as between BMI and demographic variables such as gender and age.

### Statistical analyses

We hypothesized that a higher RFEI would be associated with unhealthy dietary patterns and therefore a higher BMI particularly affecting low-income populations in urban areas of Mexico. Multilevel linear regression models were used to assess the relationship between the RFEI and dietary patterns and the RFEI and BMI. Building on the methodology discussed in previous publications [4, 19], these models employed directed acyclic graphs (DAGs) to map out presumed relationships among variables [19]. DAGs are graphical tools that depict the directional flow of these relationships without any cycles, thereby clarifying the dependency structure among multiple factors. This approach is instrumental in suggesting potential associations. DAGs facilitate the identification of the most plausible relationships between observed data patterns. By utilizing DAGs, our analysis adopted a structured framework to explore the complex interactions between variables, enhancing our capacity to draw informed and robust conclusions regarding their directionality and influence [19]. For the models, the dependent variable was either the dietary patterns or BMI. The independent variables were age, gender, household SEP (a derived index provided in the ENSANUT dataset, categorized into quintiles), physical activity (inactive, moderately active, active), car ownership (owns, does not own a car), region (north, centre, metropolitan areas, south), neighbourhood deprivation (low, high), urbanicity (rural, urban, metropolitan), food assistance program (participates in a food program, does not) and healthcare service (registered user, not a registered user).

The use of the DAGs led to the development of four statistical models ─ Models A, B, C and D [4]. For descriptive analyses and statistical models A, C and D, we considered the analytical sample of n = 13,718 individuals. For Model B, where the ’car ownership’ variable was collected only from a subsample of the population, the analytical sample was n = 13,699. Model A was adjusted for age, gender, and SEP (n = 13,718) and considered s*tate* as a second-level random effect. Model B was adjusted for variables in Model A and car ownership, neighbourhood deprivation, food assistance programs, and healthcare service. *CTA* was considered as a random effect level (n = 13,699). Model C considered Model A plus deprivation and urbanity of CTA (n = 13,718) and relied on *state* as a second-level random effect. Finally, for a sensitivity analysis, we implemented Model D, a linear regression model as advised by our Directed Acyclic Graph (DAG) test. This model attempts to mitigate selection bias by incorporating variables used in the participant selection survey, alongside additional factors such as neighbourhood deprivation, food assistance programs, and healthcare services (n = 13,718). However, it is important to recognize that while these adjustments can reduce the impact of selection bias, they cannot entirely eliminate it in an observational study. Details of diagnostics tests for all models are specified in previous publications [4, 19]. In sum, multicollinearity and Likelihood ratio and interclass correlation were tested, and models passed the tests [4, 19]. Survey design was used for all descriptive analysis and for Model D. Missing values were dropped from the analyses using the complete case analysis method [4]. When testing the association of BMI and the RFEI, interactions were also tested for the RFEI, gender and age.

## Results

### Descriptive analyses

Table 1 shows the distribution of the analytical sample of urban participants for this study, drawn from the ENSANUT 2012 survey, categorised by BMI and sociodemographic, economic and health variables used in the analyses. The descriptive analyses of this data revealed a nuanced distribution of BMI categories across different demographic and socioeconomic variables. The study found 1% underweight, 25% healthy weight, 38% overweight, and 35% obese individuals among the 13,718 participants. Gender differences were noted, with men more likely to be overweight, whilst obesity rates were higher among women. Age showed a trend where younger adults (18–24) had higher proportions of healthy weight, and obesity rates increased with age. Physical activity levels, car ownership, and region were also correlated with BMI categories, highlighting the complex interplay of lifestyle, economic, and environmental factors in obesity prevalence. In addition, 73% did not own a car compared with 56% of households nationally who do not own a car [26] (Table 1).

**Table 1:** Sociodemographic, economic, and anthropometric characteristics of the analytical sample of adult participants, from the 2012 ENSANUT survey, inhabiting urban areas of Mexico.

Variable	Underweight*	Healthy weight*	Overweight*	Obese*	Total N (%)
	N (%)	N (%)	N (%)	N (%)	
**All**	150 (1)	3,319 (25)	5,244 (38)	5,005 (35)	13,718 (100)
**Gender**					
Men	60 (1)	1,510 (26)	2,409 (42)	1,823 (31)	5,802 (100)
Women	90 (1)	1,809 (24)	2,835 (35)	83,182 (39)	7,917 (100)
**Age**					
18–24	37 (2)	695 (49)	439 (30)	276 (18)	1,447 (100)
25–34	42 (2)	830 (30)	1,038 (38)	889 (31)	2,799 (100)
35–44	17 (0)	586 (18)	1,338 (41)	1,383 (41)	3,324 (100)
45–54	11 (1)	404 (17)	975 (38)	1,144 (45)	2,534 (100)
55–64	10 (1)	292 (17)	715 (42)	731 (40)	1,748 (100)
65+	33 (2)	512 (28)	739 (41)	582 (30)	1,866 (100)
**Physical activity**					
Active	8 (1)	142 (21)	246 (36)	274 (42)	670 (100)
Moderately active	4 (1)	103 (26)	167 (36)	178 (38)	452 (100)
Inactive	22 (1)	643 (26)	1,005 (38)	946 (35)	2,616 (100)
Missing	116 (1)	2,431 (24)	3,826 (38)	3,607 (36)	9,980 (100)
**Household SEP** ^**††**^					
Highest	25 (2)	400 (29)	507 (39)	436 (31)	1,368 (100)
Second highest	26 (1)	529 (26)	871 (39)	791 (34)	2,217 (100)
Middle	34 (2)	710 (26)	1,081 (36)	1,121 (36)	2,946 (100)
Second lowest	38 (1)	759 (23)	1,311 (39)	1,288 (37)	3,396 (100)
Lowest	27 (1)	921 (25)	1,474 (39)	1,369 (35)	3,791 (100)
**Car ownership**					
Owns a car	22 (1)	885 (25)	1,483 (40)	1,346 (34)	3,736 (100)
Does not own a car	128 (1)	2,429 (25)	3,753 (38)	3,653 (36)	9,963 (100)
Missing	1 (0)	5 (26)	8 (42)	6 (32)	20 (100)
**Region**					
South	46 (1)	1,249 (24)	2,142 (40)	2,062 (36)	5,499 (100)
North	38 (2)	599 (25)	913 (36)	992 (38)	2,542 (100)
Centre	61 (1)	1,393 (27)	2,041 (38)	1,812 (34)	5,307 (100)
Metropolitan area	5 (1)	78 (23)	148 (39)	139 (36)	370 (100)
**Deprivation**					
Low	128 (1)	2,839 (25)	4,407 (38)	4,271 (36)	11,645 (100)
High	22 (1)	480 (25)	837 (42)	734 (32)	2,073 (100)
**Food Program**					
Participates	120 (1)	2,815 (25)	4,522 (39)	4,271 (35)	11,728 (100)
Does not participate	30 (1)	504 (27)	722 (35)	734 (37)	1,990 (100)
**Healthcare service**					
Registered user	45 (2)	823 (29)	1,067 (36)	1,019 (34)	2,954 (100)
Not a registered user	105 (1)	2,496 (24)	4,177 (39)	3,986 (36)	10,764 (100)

*Body mass index (BMI): Underweight: 15 to <18.5 kg/m^2^; healthy weight: ≥18.5 to < 25 kg/m^2^; overweight: 25 to < 30 kg/m^2^; obese: ≥30 to 58 kg/m^2^, n = 13,718.

^**††**^ Quintiles

In the urban census tract areas of Mexico, the mean RFEI was 17.89 (SD = 12.41), with values ranging from 0.70 to 98.00. This indicates substantial variability in the distribution of fast-food outlets, convenience stores compared to fruit and vegetable stores and supermarkets across the urban areas. The descriptive statistics provided insight into the diversity of the food environment, with some census tracts exhibiting a higher concentration of fast-food outlets and convenience stores compared to fruit and vegetable stores and supermarkets. Additionally, there were 9,205 census tracts with no food outlets, emphasizing the heterogeneity in food availability across urban areas (Table 2).

**Table 2:** Retail Food Environment Index in urban census tract areas in Mexico.

Outcome	Mean density (SD)	Min	Max	Q1	Q2	Q3	Number of CTAs with no food outlets
RFEI	17.89 (12.41)	0.70	98.00	6	11	17	9,205 (16.61%)

RFEI: The Retail Food Environment Index quantifies the balance between fast-food outlets and convenience stores divided by supermarkets and fruit and vegetable stores within a given area, specifically a census tract area (CTA). It calculates this balance by dividing the sum of fast-food outlets and convenience stores by the sum of fruit and vegetable stores plus supermarkets. This metric helps in assessing the potential availability of healthy versus unhealthy food choices in urban environments.

SD: standard deviation.

Min: minimum density of food outlets per census tract area.

Max: maximum density of food outlets per census tract area.

Mean density: the average RFEI value across all CTAs, indicating the ratio of fast-food outlets and convenience stores to supermarkets and fruit and vegetable stores.

Percentiles (Q1, Q2, Q3): represent the RFEI distribution across census tract areas (CTAs), providing insights into the range and typical values. Q1 (25th percentile) indicates that 25% of CTAs have an RFEI below this value, showing lower availability of fast-food outlets and convenience stores compared to supermarkets and fruit and vegetable stores. Q2 (50th percentile or median) reflects the middle value, and Q3 (75th percentile) means 75% of CTAs have an RFEI below this value, highlighting areas with a higher concentration of fast-food outlets and convenience stores.

CTA: Census tract area.

Total number of urban CTAs = 55,427. CTAs with no food outlets represent areas lacking immediate access to both healthy and unhealthy food options, underscoring potential food deserts. This absence can significantly impact residents’ dietary choices, possibly leading to increased reliance on distant food sources or less nutritious, non-perishable foods. Understanding these areas helps in identifying and addressing gaps in food accessibility and planning interventions to improve public health nutrition.

To categorize the RFEI into distinct groups indicating low, moderate, and high levels, we used a quantile categorization approach. Specifically, we defined the categories as follows: a low RFEI corresponded to values within the 0-25th percentile range, a moderate RFEI included values from the 26th to the 75th percentile, and a high RFEI encompassed values in the 76th to 100th percentile range (Table 2).

### Retail Food Environment Index and BMI

We found that Model A had the highest and therefore best Interclass Correlation Coefficient (ICC) (0.11 SE: 00.2 95%CI: 0.07, 0.17). Model A, which was adjusted for age, gender, and SEP, showed that a higher RFEI was associated with a higher BMI (β = 0.010, 95% CI: 0.0005, 0.02, p = 0.04). Similarly, Models B, C and D also showed a statistically significant association between the RFEI and BMI. An effect size of β = 0.010 was found, which indicates that a 1-unit increase in the RFEI was associated with a BMI increase of 0.010 kg/m^2^ (Table 3). A unit increase in RFEI indicates a higher prevalence of fast-food outlets and convenience stores relative to supermarkets and fruit and vegetable stores in a CTA, suggesting an increased potential exposure to food outlets that tend to offer foods high in saturated fat, salt and sugar.

**Table 3:** Multilevel linear regression models of Retail Food Environment Index and body mass index.

Model	β (95% CI)	p-value
Model A	**0.010 (0.0005, 0.020)**	**0.040**
Model B	**0.012 (0.0004, 0.020)**	**0.043**
Model C	0.010 (-0.0001, 0.020)	0.053
Model D	**0.014 (0.0006, 0.030)**	**0.040**

All results indicate coefficients (β) and confidence interval (CI) in parenthesis. β represents the increase of BMI in kg/m^2^ per every unit increase of the Retail Food Environment Index, n = 13,718.

### Retail Food Environment Index and dietary patterns

The application of multilevel linear regression models enabled an exploration of the relationship between the RFEI and dietary patterns. This analysis showed an association, particularly with the unhealthy dietary pattern. Factor 2 shown in S3 Text provides a comprehensive view, encompassing various food groups laden with saturated fats, such as sausages, sugar-laden desserts, dressings, soda, fast-food items, refined cereal, ready-to-eat soups, and indulgent snacks such as potato chips and candy. These food items display positive factor loadings (≥0.30), underscoring their strong association with the unhealthy dietary pattern. The subsequent statistical analysis yielded a statistically significant β coefficient of 0.100 (95% CI: 0.03, 0.12, p = 0.001) for the unhealthy dietary pattern. This implies that for every one-unit increase in the RFEI, there is a corresponding increase of 0.100 in the score of the unhealthy dietary pattern. These dietary pattern scores are standardized z-scores, meaning they have a mean of 0 and a standard deviation of 1, allowing for comparison across different patterns and indicating relative adherence to the unhealthy dietary pattern (Table 4).

**Table 4:** Multilevel linear regression model of Retail Food Environment Index and dietary patterns in Mexico.

Dietary pattern	β (95% CI)	p-value
Healthy dietary pattern	0.005 (-0.05, 0.06)	0.858
Unhealthy dietary pattern	**0.100 (0.03, 0.12)**	**0.001**
Carbohydrate dietary pattern	0.002 (-0.06, 0.06)	0.949

β represents the association of dietary patterns and the Retail Food Environment Index.

Regression analysis was undertaken considering Model A (adjusted for age, gender, SEP to test the association of dietary patterns (outcome variable) and the RFEI (exposure variable), n = 1,572.

### Retail Food Environment Index and socioeconomic position

The findings outlined in Table 5 present our statistical analysis of the SEP and its correlation with the RFEI in urban CTAs throughout Mexico. The association of SEP (outcome) with RFEI (exposure) using multilevel logistic regression showed a significant negative correlation between SEP and the RFEI. The odds ratio (OR) for SEP was 0.97 (95% CI: 0.97 to 0.97, p < 0.001), indicating that for each increment in SEP, the odds of being exposed to a food environment with a higher level of fast-food outlets and convenience stores and decreases by 3%, suggesting that individuals from lower socioeconomic backgrounds are likely to live in neighbourhoods with higher RFEI scores, indicative of increased exposure to retail food environments that offer unhealthy food options (Table 5).

**Table 5:** Multilevel linear regression models of Retail Food Environment Index and socioeconomic position in Mexico, adjusted for age and gender.

Variable	OR (95% CI) ^†^	p-value	β (95% CI) ^††^	p-value	β (95% CI) ^†††^	p-value
Socioeconomic position	**0.97 (0.97, 0.97)**	**<0.001**				
Socioeconomic position (Quintiles)						
Higher upper				Ref	Ref	
Upper middle			0.007 (-0.01, 0.02)	0.374	**0.02 (0.02, 0.03)**	**<0.001**
Middle			0.005 (-0.01, 0.02)	0.509	**0.04 (0.03, 0.04)**	**<0.001**
Upper lower			0.004 (-0.01, 0.02)	0.684	**0.05 (0.04, 0.05)**	**<0.001**
Lower			**0.02 (0.006, 0.04)**	**0.010**	**0.06 (0.05, 0.07)**	**<0.001**

† Multilevel logistic regression. Results indicate odds ratios (OR) and confidence interval (CI) in parenthesis; n = 13,718.

†† Multilevel linear regression, association of the RFEI (exposure) and BMI (outcome) stratified by socioeconomic position quintiles. Results indicate coefficients (β) and confidence interval (CI) in parenthesis. β represents the increase of SEP per every unit increase of the Retail Food Environment Index. n = 13,718.

††† Generalised structural equation models which results indicate coefficients (β) and confidence interval (CI) in parenthesis; n = 13,718.

Regarding the multilevel linear regression, which tested the association between BMI (outcome) and the RFEI (exposure), stratified by SEP quintiles, showed that households with lower SEP had higher RFEI scores compared to those from higher SEP households (β = 0.020, 95% CI: -0.006 to 0.04, p = 0.10). Similarly, when looking at the generalised structural equation models, a graded relationship between RFEI scores and SEP was observed indicating that higher SEP households had lower RFEI scores *(*β = 0.02, 95% CI: 0.02, 0.03, p < 0.001) whilst lower SEP households were exposed to higher RFEI scores (β = 0.060, 95% CI: 0.05 to 0.07, p < 0.001) (Table 5).

Finally, the research sought to identify any interactions between SEP and RFEI, as well as between BMI and demographic factors such as gender and age. The analyses, reflected in S4 Text, found no statistically significant interactions, indicating that the linkage between SEP and RFEI remains stable across various demographic groups.

## Discussion

Our investigation of the association between the RFEI, BMI, dietary patterns, and SEP in urban areas of Mexico confirms that food environments with an abundant access of fast-food outlets and convenience stores are associated with higher BMI and poorer dietary intake. This study enhances our understanding of the factors that have an association with obesity-related outcomes.

### Association between RFEI, BMI, and dietary patterns

The RFEI served as a measure of the food environment. Individuals residing in CTAs with a higher RFEI, or a greater proportion of fast-food outlets and convenience stores were more likely to exhibit higher BMI values and adopt unhealthy dietary patterns. Consequently, neighbourhoods characterized by a higher density of fast-food outlets and convenience stores may elevate the risk of residents consuming diets associated with increased BMI and a higher incidence of non-communicable diseases. This association may be due to the high level of HFSS foods commonly offered in these types of stores which can lead to higher BMI levels [27]. To put this into perspective, for an individual weighing 60 kg and standing 1.60 m tall, the β coefficient of 0.010, which represents the increase in BMI (kg/m^2^) for each unit increase in the RFEI, translates to a potential weight gain of 0.032 kg. This is associated with each additional convenience store and fast-food outlet in their neighbourhood compared to the number of supermarkets and fruit and vegetable stores per square kilometre. For a 10% higher RFEI, this would result in an approximate weight gain of 0.046 kg. Although the β coefficient of 0.010 was statistically significant, the clinical significance of the association between RFEI and BMI might seem minimal at the individual level. However, when considering obesity prevention at the population level, even small increases in weight can be important. This interpretation highlights the need to consider the broader impact of food environments on public health and the importance of multifaceted approaches to obesity prevention.

The analysis of the association between the RFEI and dietary patterns indicated that individuals residing in areas characterized by a higher RFEI were more likely to adopt dietary habits featuring the consumption unhealthy dietary patterns, indicating a significant association between the food environment and dietary choices. The findings suggest that higher RFEI values, which represent a higher ratio of fast-food outlets and convenience stores compared to supermarkets and fruit and vegetable stores, are significantly associated with greater adherence to unhealthy dietary patterns. This highlights the potential impact of the retail food environment on dietary behaviours and underscores the importance of considering food environment factors in public health interventions aimed at improving diet quality.

The approach of the RFEI, helps to understand the balance between outlets, which in their majority may sell predominantly unhealthy or healthier alternatives and combines the concepts of food deserts (i.e., limited availability of food outlets that have a higher offering of fruits and vegetables and other nutrient-dense foods) and food swamp (i.e., increased availability of food outlets with a higher offer of HFSS foods) into a single measure, providing a nuanced understanding of how the overall food environment may be associated with dietary patterns and BMI. The observed association between RFEI and unhealthy dietary patterns adds another layer to the complexity of factors contributing to obesity-related outcomes. However, it is crucial to recognize that whilst the RFEI serves as a valuable metric for assessing the food environment, individual dietary choices within this environment play a substantial role in shaping health outcomes.

### Association between RFEI and SEP

Concerning socioeconomic disparities, CTAs with a higher RFEI (i.e., higher proportion of fast-food outlets and convenience stores) were more often located where there were more households of lower SEP. Previous studies have identified a link between higher exposure to obesogenic food environments in low-income neighbourhoods [24, 28]. Low income areas have been found to be saturated with food stores, of which most offer unhealthy food options [29]. Exposing lower SEP households to a greater availability of the SSB and ultra-processed foods places this sector of the population at a greater risk for obesity [4, 24].

The findings of this study concur with those from higher-income countries, where a greater availability of fast-food outlets or convenience stores, which tend to provide high accessibility of SSB, and ultra-processed foods, has been linked with a higher BMI [27, 30–34]. Other studies have also found a correlation between higher RFEI (or similar indices) and higher BMI [35, 36].

### Strengths and limitations

This study acknowledges certain limitations that warrant discussion. The use of cross-sectional data, a common constraint in many studies, precludes an exploration of temporality and prohibits making causal inferences. It is crucial to note that the strategic availability of fast-food outlets and convenience stores (higher RFEI) in locations conducive to sales success or populated by individuals with higher BMIs may introduce biases, potentially affecting the observed associations [4]. A notable limitation arises from the temporal disparity between the collection of health data and geographic data. Health-related information was gathered two years prior to the acquisition of food outlet data, introducing the possibility of changes in the food environment during this interval. Existing literature highlights the common occurrence of variability in the temporal alignment of food store data and health-related information, and differences in data availability from government or commercial sources [37–40]. This two-year gap may have led to discrepancies due to urban development changes, as evidenced by our ground-truthing findings. During the ground-truthing, conducted by the lead author after completing specialized training, nine randomly sampled areas in Hermosillo were assessed. While 84% of the food outlets from the 2014 INEGI dataset matched the actual locations verified in 2016, the 16% discrepancy highlights the dynamic nature of urban environments and the potential for changes over time. Additionally, the ground-truthing was only performed in one city, which may limit the generalizability of our findings to other urban areas in Mexico. Despite these limitations, the ground-truthing process is a strength of our study, providing a more accurate and up-to-date representation of the food environment. By incorporating these on-the-ground verifications, we were able to identify informal food establishments not previously recorded, thereby enriching our understanding of the local food landscape. This methodological approach enhances the reliability of our findings, though caution is warranted when interpreting the results due to the noted temporal and geographic limitations (S1 Text).

Another limitation stems from the lack of exact geographical coordinates for individuals’ locations. To maintain survey participant confidentiality, outcome data were computed based on the centre of individuals’ census tract areas (CTAs) [4]. Whilst this approach safeguards privacy, it introduces a degree of spatial imprecision. Spatial dependency, a crucial consideration in food environment studies, was not explicitly assessed in this study. Most studies often employ non-spatial statistical approaches, assuming observations are independent and that relationships among variables are stationary [41]. However, geographic proximity among observations may result in shared characteristics, potentially introducing bias. Future investigations should incorporate spatial dependence analysis, such as spatial autocorrelation, to evaluate whether residuals from regression analyses exhibit spatial autocorrelation [42]. Addressing these limitations enhances the robustness and applicability of findings in understanding the nuanced relationship between the food environment and health outcomes.

The use of the RFEI as a main measure of the food environment in this study poses potential limitations. The RFEI was not validated for use in low- and middle-income countries such as Mexico; however, food outlets were classified to align with the categories used in the RFEI. The RFEI focuses only on the geographic distribution of food retailers but does not account for human factors, such as mobility and other sociodemographic factors, which impact the influence of the food environment on a population’s diet [43].

Furthermore, this study did not assess the healthfulness of food stores. There may be fast-food outlets or convenience stores that predominantly sell healthy foods, which would require an in-store food assessment for a more precise measure of the RFEI. Whilst our study focused on assessing the association between the RFEI and obesity-related outcomes, it is important to consider the in-store food environment, which may play a significant role in shaping individual food choices and, subsequently, obesity [44–47]. Factors such as the availability of affordable and healthy alternatives, and the placement and promotion of food products within stores, could have a substantial impact on food choices [44–47]. Although these aspects were not within the scope of our current investigation, acknowledging their potential association with dietary intake and obesity is important.

Lastly, a reduced sample size for dietary intake, car ownership and physical activity is a limitation of the nutrition and health survey used (ENSANUT 2012) and may affect the power to show significant associations. In addition, due to the exclusion of participants inhabiting rural areas, the availability of complete and accurate data, and to some individuals not having a RFEI (geographic) is that we had a reduced sample size (n = 13,718). Whilst efforts were made to maximize data collection, these gaps exist due to logistical constraints, participant compliance, or specific data collection limitations in certain regions or among certain subgroups. Despite these challenges, we have thoroughly addressed the available data in our analyses and interpretations. To account for this limitation, we carried out sensitivity analyses using statistical models that excluded these variables.

Regarding the strengths of this study, this research assessed the relationship between the RFEI, BMI, dietary patterns, and SEP within a middle-income country. Building on from previous research [4], it added the study of dietary patterns as an outcome, the RFEI as an exposure, and various measures of SEP. Additionally, the use of the RFEI is a particular strength as it offers a holistic perspective on the food environment. Rather than isolated assessments of individual food outlets, the RFEI provided an overall pattern of the food environment [48]. This approach provides a nuanced understanding of how the overall food environment relates to dietary patterns and health outcomes.

### Policy implications

The association between the RFEI, BMI, dietary patterns, and SEP in urban areas of Mexico provides relevant insights for public health policies. Targeted interventions are warranted to address obesogenic environments, emphasizing policies that increase the availability and affordability of healthy food options whilst restricting access to unhealthy alternatives [49]. Drawing inspiration from successful initiatives worldwide, policymakers can consider strategies such as the ’Healthier Hawker Food Programme’ in Singapore, supporting food vendors to provide healthier options, or the ’Healthy Food Financing Initiative’ in the United States, which provides grants to attract healthier retail outlets to underserved areas [50]. Examples from various countries, including San Francisco’s Health Food Incentives Ordinance and New York’s ’Green Cart Permit,’ [51]. showcase the potential of regulatory measures to shape dietary patterns [52]. France’s ban on unlimited offers of sweetened beverages in specific settings [53] and Scotland’s ’Healthy Living Neighbourhood Shops’ project, focusing on improving the availability of healthy items [54], exemplify diverse approaches that, when combined, can contribute to fostering healthier access to food and combating obesity.

## Conclusion

Our study highlights the significant association of the RFEI on BMI, dietary patterns, and SEP in urban Mexico. We found that a higher RFEI, indicative of a higher number of fast-food outlets and convenience stores, correlates with a higher BMI and unhealthy dietary patterns, particularly affecting lower SEP households. This underscores the need for public health policies to create more supportive environments for healthy eating, considering the multifaceted factors contributing to obesity.

## Supporting information

S1 Text Ground-truthing. (DOCX)

S2 Text Diagnostic test. (DOCX)

S3 Text Dietary patterns. (DOCX)

S4 Text Socioeconomic position interactions. (DOCX)
