# Supplementary material for: The Retail Food Environment Index and its association with dietary patterns, body mass index, and socioeconomic position: A multilevel assessment in Mexico
Source: PLOS Glob Public Health. 2024 Oct 10;4(10):e0003819. doi: 10.1371/journal.pgph.0003819 (PMC11466391; doi:10.1371/journal.pgph.0003819)
Supplement: S1 Text — (DOCX) [file pgph.0003819.s001.docx]

**The Retail Food Environment Index and its association with dietary patterns, body mass index, and socioeconomic position: a multilevel assessment in Mexico**

**Supporting Information**

# S1. Ground-truthing

## Ground-truthing Background

Ground-truthing is a methodology utilized to validate the existence and geolocation of buildings, landmarks, or land areas. Satellite images, along with sources like the Yellow pages and governmental geographical datasets, serve as references for comparison with actual ground locations ([GISGeography, 2023](https://gisgeography.com/ground-truthing/)). Defined by the Environmental Systems Research Institute (ESRI) as the accuracy of remotely sensed or mathematically calculated data based on field measurements ([ESRI, 2021](https://desktop.arcgis.com/en/arcmap/latest/manage-data/raster-and-images/accuracy-assessment-for-image-classification.htm)), ground-truthing aims to provide a precise understanding of an area's functionality and structure. This process ensures the data being analysed, particularly concerning the existence of food outlets, is accurate and devoid of errors during analysis ([ESRI, 2021](https://desktop.arcgis.com/en/arcmap/latest/manage-data/raster-and-images/accuracy-assessment-for-image-classification.htm)).

## Ground-truthing Methods

To verify the existence and locations of food outlets, including convenience stores, fast-food outlets, restaurants, and supermarkets, the city of Hermosillo, Mexico, was selected. Hermosillo, the capital of Sonora, is divided into 346 census tract areas (CTAs) in its urban region. The selection involved randomly choosing nine areas, each from a cardinal direction (North, South, West, and East). Symbols were assigned to different types of food establishments based on their characteristics and food offerings, and these symbols were mapped according to the geolocation of the food outlets.

Verification activities were conducted on foot and by car, with routes designed for optimal observation of food outlets. Geolocation and existence were confirmed through pictures taken during the process. The selected areas varied by socioeconomic position (SEP), influencing road quality, crime levels, and the types of food establishments available.

The ground-truthing findings revealed an increase in supermarkets and convenience stores, with a notable presence of informal food establishments, such as street food stands, not previously verified in the INEGI database. Continuous growth was observed in both supermarkets and convenience stores, while restaurants and locally owned/non-chain convenience stores showed changes in geolocation in several instances.

In summary, the ground-truthing process indicated that 84% of the INEGI dataset from 2014 aligned with the actual geolocation of food outlets verified in 2016. The 16% discrepancy was attributed to urban development leading to changes in the retail food environment over time. Consequently, considering the temporal variation, caution is advised when interpreting the results of our study on the food environment and its association with BMI in Mexico.

**Fig A. Ground-truthing: Example of map used of sampled areas in Mexico with food outlet geolocation registered by INEGI and an overlaid field verification of the food outlets and their location.**

Δ symbol denotes a geolocated food outlet that has been both identified and verified using the 2014 database from the Instituto Nacional de Estadística y Geografía (INEGI), Mexico. The base layer of this map represents the country of Mexico, constructed with ArcGIS 10.2.2 (ESRI, Redlands, CA) software. The foundational basemap shapefile is derived from 'INEGI.' For detailed source information, refer to: Mexico: Instituto Nacional de Estadística y Geografía; 2017. Citation as of September 25, 2017. Accessible at: <https://www.inegi.org.mx/app/mapas/>
