# Supplementary material for: The Retail Food Environment Index and its association with dietary patterns, body mass index, and socioeconomic position: A multilevel assessment in Mexico
Source: PLOS Glob Public Health. 2024 Oct 10;4(10):e0003819. doi: 10.1371/journal.pgph.0003819 (PMC11466391; doi:10.1371/journal.pgph.0003819)
Supplement: S2 Text — (DOCX) [file pgph.0003819.s002.docx]

**The Retail Food Environment Index and its association with dietary patterns, body mass index, and socioeconomic position: a multilevel assessment in Mexico**

**Supporting Information**

# **S2. Diagnostic Tests**

**Fig B. Histogram of BMI normality distribution test**

The figure presents the histogram for BMI normality distribution.

**Heteroscedasticity test**

Models were tested for heteroscedasticity.

**Fig C. Heteroscedasticity test**


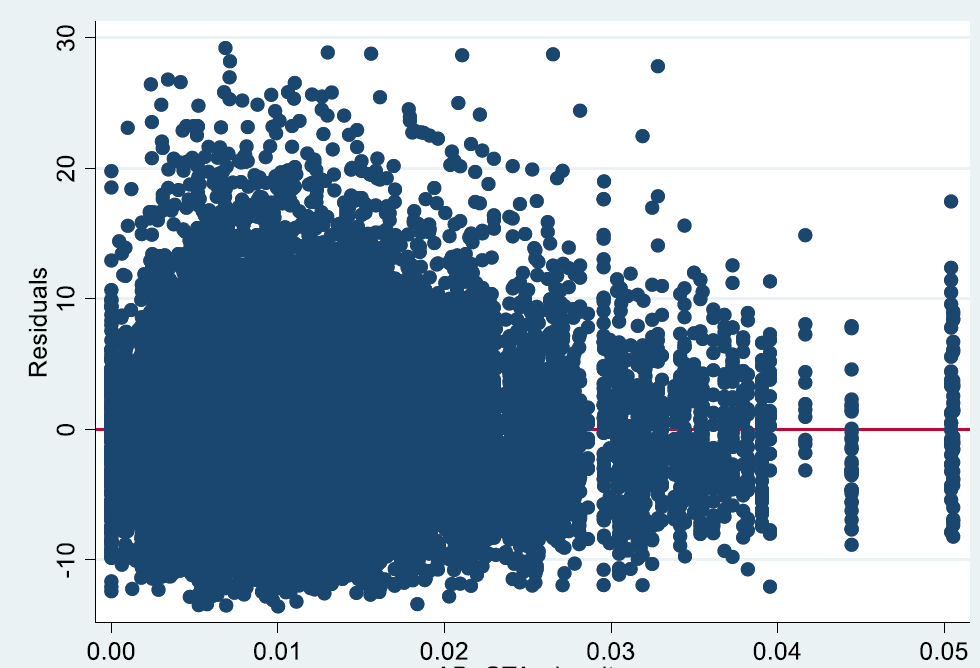


The test shows the relationship between residuals and the density of food outlets.
