# Supplementary material for: The Retail Food Environment Index and its association with dietary patterns, body mass index, and socioeconomic position: A multilevel assessment in Mexico
Source: PLOS Glob Public Health. 2024 Oct 10;4(10):e0003819. doi: 10.1371/journal.pgph.0003819 (PMC11466391; doi:10.1371/journal.pgph.0003819)
Supplement: S3 Text — (DOCX) [file pgph.0003819.s003.docx]

**The Retail Food Environment Index and its association with dietary patterns, body mass index, and socioeconomic position: a multilevel assessment in Mexico**

**Supporting Information**

# **S3. Dietary patterns**

| **Table A. Dietary patterns, factor loadings, proportion of variance explained and Cronbach’s alpha** | | | | |  |
| --- | --- | --- | --- | --- | --- |
|  | **Food groups** | **Factor 1** | **Factor 2** | **Factor 3** |  |
|  |  |  |  |  |  |
|  |  | **Healthy pattern** | **Unhealthy pattern** | **Carbohydrate pattern** |  |
| 1 | Vegetables | **0.718** | 0.062 | 0.057 |  |
| 2 | Lemon and chili | **0.526** | 0.224 | -0.255 |  |
| 3 | Meat (e.g. pork, beef, chicken) | **0.494** | 0.193 | 0.053 |  |
| 4 | Cooked meals (e.g. rice, stews with vegetables) | **0.478** | 0.047 | 0.090 |  |
| 5 | Fruit | **0.446** | -0.037 | **0.424** |  |
| 6 | Bread, crackers, and potatoes | **0.431** | 0.136 | 0.019 |  |
| 7 | Soups | **0.426** | 0.040 | 0.082 |  |
| 8 | Fermented dairy (cheese and yogurt) | **0.344** | 0.271 | 0.241 |  |
| 9 | Fats | **0.314** | **0.330** | -0.110 |  |
| 10 | Juice and natural drinks | 0.296 | -0.071 | 0.360 |  |
| 11 | Fried beans and pulses | 0.285 | 0.240 | -0.123 |  |
| 12 | Fish and seafood | 0.276 | 0.017 | 0.152 |  |
| 13 | Beans and pulses in water | 0.248 | -0.241 | -0.184 |  |
| 14 | Coffee (with and without sugar) | 0.232 | -0.053 | -0.214 |  |
| 15 | High meat and fatty meals | 0.207 | 0.286 | -0.070 |  |
| 16 | Tea (with and without sugar) | 0.199 | -0.122 | 0.036 |  |
| 17 | Sugar and desserts | 0.189 | **0.511** | **0.337** |  |
| 18 | Sausages | 0.188 | **0.439** | 0.036 |  |
| 19 | Water | 0.172 | -0.042 | 0.020 |  |
| 20 | Whole wheat products | 0.126 | -0.021 | **0.330** |  |
| 21 | Milk | 0.090 | 0.110 | **0.629** |  |
| 22 | Alcohol | 0.080 | 0.147 | -0.254 |  |
| 23 | Dressings | 0.021 | **0.593** | 0.018 |  |
| 24 | Soda | 0.014 | **0.589** | -0.172 |  |
| 25 | Fast-food (burger, pizza, hot-dog) | -0.009 | **0.547** | 0.204 |  |
| 26 | Refined cereal | -0.017 | 0.215 | 0.531 |  |
| 27 | Ready-to-eat soups | -0.043 | **0.388** | -0.006 |  |
| 28 | Potato chips and candy | -0.081 | **0.644** | 0.086 |  |

A value of >0.30 indicates that food group is associated with a factor. Bold factors indicate an association. Note: The values in the table represent factor loadings, which are coefficients that indicate the strength and direction of the relationship between each food group and the identified dietary patterns (Factor 1, Factor 2, Factor 3). These factor loadings are dimensionless, meaning they do not have specific units. Factor analysis is a statistical method used to identify patterns and relationships among variables, and in this context, the loadings signify the extent to which each food group contributes to a particular dietary pattern. The absolute value of the factor loading indicates the strength of the association, with values greater than 0.30 considered to have a meaningful contribution to the respective factor. The factors, represented by the Healthy pattern, Unhealthy pattern, and Carbohydrate pattern, serve as composite variables derived from the combination of the included food groups based on their loadings.
