# Supplementary material for: The Retail Food Environment Index and its association with dietary patterns, body mass index, and socioeconomic position: A multilevel assessment in Mexico
Source: PLOS Glob Public Health. 2024 Oct 10;4(10):e0003819. doi: 10.1371/journal.pgph.0003819 (PMC11466391; doi:10.1371/journal.pgph.0003819)
Supplement: S4 Text — (DOCX) [file pgph.0003819.s004.docx]

**The Retail Food Environment Index and its association with dietary patterns, body mass index, and socioeconomic position: a multilevel assessment in Mexico**

**Supporting Information**

# **S4. Socioeconomic position interactions**

**Table B. Interaction between the retail food environment index (RFEI) and socioeconomic position when considering the association between BMI and the RFEI**

| SEP | β coefficient (95%CI) | p-value |
| --- | --- | --- |
| Model A |  |  |
| Higher Upper | Ref |  |
| Upper Middler | -0.007 (-0.03, 0.02) | 0.655 |
| Middle | -0.02 (-0.05, 0.01) | 0.264 |
| Upper lower | -0.02 (0.05, 0.001) | 0.184 |
| Lower | 0.001 (-0.03, 0.03) | 0.926 |
| Model B |  |  |
| Higher Upper | Ref |  |
| Upper middle | -0.004 (-0.03, 0.03) | 0.809 |
| Middle | -0.01 (-0.04, 0.049) | 0.491 |
| Upper lower | -0.01 (-0.04, 0.02) | 0.398 |
| Lower | 0.006 (-0.03, 0.04) | 0.718 |
| Model C |  |  |
| Higher Upper | Ref |  |
| Upper middle | -0.006 (-0.03, 0.02) | 0.694 |
| Middle | -0.02 (-0.04, 0.01) | 0.281 |
| Upper lower | -0.02 (-0.05, 0.01) | 0.195 |
| Lower | 0.002 (-0.03, 0.03) | 0.914 |
| Sensitivity model |  |  |
| Higher Upper | Ref |  |
| Upper middle | 0.02 (-0.02, 0.05) | 0.348 |
| Middle | 0.001 (-0.03, 0.03) | 0.937 |
| Upper lower | 0.009 (-0.02, 0.04) | 0.581 |
| Lower | 0.04 (-0.01, 0.10) | 0.147 |

All results indicate coefficients (β) and confidence interval (CI) in parenthesis. β represents the increase of BMI in kg/m^2^ per every unit increase of the Retail Food Environment Index (RFEI).

Model A: Age, gender, and socioeconomic position; n=13,718.

Model B: Model A + car ownership, neighborhood deprivation, food assistance programs, health insurance, and socioeconomic position, CTA (2nd level); n=13,699.

Model C: Model A + deprivation and urbanity of CTA; n=13,718

Model D: Model A + neighborhood deprivation, food assistance programs, health insurance and socioeconomic position; n=13,718.

**Table C. Interaction between the RFEI and gender when considering the association of BMI and the RFEI**

| SEP | β coefficient (95%CI) | p-value |
| --- | --- | --- |
| Model A |  |  |
| Men | Ref |  |
| Women | 0.006 (-0.013, 0.025) | 0.549 |
| Model B |  |  |
| Men | Ref |  |
| Women | 0.006 (-0.012, 0.025) | 0.515 |
| Model C |  |  |
| Men | Ref |  |
| Women | 0.006 (-0.013, 0.025) | 0.539 |
| Sensitivity model |  |  |
| Men | Ref |  |
| Women | 0.005 (-0.17, 0.03) | 0.681 |

All results indicate coefficients (β) and confidence interval (CI) in parenthesis. β represents the increase of BMI in kg/m^2^ per every unit increase of the RFEI.

Model A: Age, gender, and socioeconomic position; n=13,718.

Model B: Model A + car ownership, neighborhood deprivation, food assistance programs, and health insurance, CTA (2nd level); n=13,699.

Model C: Model A + deprivation and urbanity of CTA; n=13,718

Model D: Model A + neighborhood deprivation, food assistance programs, and health insurance; n=13,718.

**Table D. Interaction between the RFEI and sex when considering the association between BMI and the RFEI**

| SEP | β coefficient (95%CI) | p-value |
| --- | --- | --- |
| Model A |  |  |
| 18-24 | Ref |  |
| 25-34 | 0.007 (-0.03, 0.04) | 0.703 |
| 35-44 | 0.01 (-0.02, 0.05) | 0.396 |
| 45-54 | 0.002 (-0.033, 0.04) | 0.913 |
| 55-64 | -0.01 (-0.05, 0.02) | 0.440 |
| 65+ | -0.007 (-0.04, 0.03) | 0.706 |
| Model B |  |  |
| 18-24 | Ref |  |
| 25-34 | 0.007 (-0.03, 0.04) | 0.670 |
| 35-44 | 0.01 (-0.02, 0.05) | 0.338 |
| 45-54 | 0.002 (-0.03, 0.04) | 0.914 |
| 55-64 | -0.02 (-0.05, 0.02) | 0.403 |
| 65+ | -0.01 (-0.05, 0.03) | 0.649 |
| Model C |  |  |
| 18-24 | Ref |  |
| 25-34 | 0.007 (-0.03, 0.04) | 0.688 |
| 35-44 | 0.01 (-0.018, 0.05) | 0.390 |
| 45-54 | 0.002 (-0.03, 0.04) | 0.895 |
| 55-64 | -0.01 (-0.05, 0.02) | 0.440 |
| 65+ | -0.007 (-0.04, 0.03) | 0.718 |
| Sensitivity model |  |  |
| 18-24 | Ref |  |
| 25-34 | 0.01 (-0.02, 0.04) | 0.579 |
| 35-44 | 0.01 (-0.03, 0.05) | 0.552 |
| 45-54 | -0.002 (-0.05, 0.05) | 0.943 |
| 55-64 | -0.03 (-0.07, 0.02) | 0.181 |
| 65+ | -0.0004(-0.04, 0.04) | 0.985 |

All results indicate coefficients (β) and confidence interval (CI) in parenthesis. β represents the increase of BMI in kg/m^2^ per every unit increase of the RFEI.

Model A: Age, gender, and socioeconomic position; n=13,718.

Model B: Model A + car ownership, neighborhood deprivation, food assistance programs, and health insurance, CTA (2nd level); n=13,699.

Model C: Model A + deprivation and urbanity of CTA; n=13,718

Model D: Model A + neighborhood deprivation, food assistance programs, and health insurance; n=13,71
